# Permanent Military Hospitals

**Published:** 1900-10-06

**Authors:** 


### The Institutional Workshop.

### PERMANENT MILITARY HOSPITALS.

### I.—GENERAL CONSIDERATIONS.

A military hospital differs from a civil hospital from the circumstance that the latter deals mainly, and it may be said wholly, with the acutely ill. In a military hospital the large majority of the inmates are convalescent, in the sense that they are able to be up and about during the greater part of each day. It is essential that this fact should be kept constantly in mind when discussing the accommodation and nursing of a military hospital. Another feature distinctive of the military hospital is that the patients are all men, mostly young men, all of them in the prime of life, full of animal spirits, and they so need a system of administration to enforce discipline and control which is altogether unnecessary in a civil hospital. For these reasons and others a great military hospital which is well organised is so arranged that a certain number of wards are set apart, in charge of the nursing sisters, to which none but the acutely ill are admitted. The rest of the wards, like those in a convalescent institution, are assigned to patients who are able to get up and spend the day in the corridors and day-rooms provided for their accommodation.

#### How to Arrange the Nursing.

We have heard a great deal of late, and are likely to hear more, in favour of an agitation for the introduction of a much greater number of female nurses into the military hospitals. It would be well for those who are moving in this direction to stay their hands until they have studied the question in all its bearings. There are sound administrative reasons why a large increase of female nurses in military hospitals would prove objectionable in practice, and be calculated to introduce evils from which our military hospitals are at present happily free. Those who are interested in the subject should make it their business to visit a place like Woolwich, where the military hospital is contiguous to a large fever hospital under the jurisdiction of the Metropolitan Asylums Board. They would there realise for themselves, especially on the days when the staffs of these two hospitals have leave of absence, why it is undesirable that the present agitation in favour of multiplying the number of female nurses employed in military hospitals should be further maintained. We have always been in favour of the Army Nursing Department, and the institution of the system calculated to secure to the fullest extent the best nursing for the really sick soldier when warded. The military authorities have shared this view, and they have succeeded in obtaining the services of some of the most efficient and best trained ladies in the nursing profession, who have done splendid service for our army in the field and in the military hospitals at home. Amongst these ladies are some of the ablest administrators in the nursing profession; they have had practical experience of the requirements and difficulties of providing for the adequate nursing of the British soldier in efficiently administered military hospitals. We venture to hope that neither the Army Medical Department nor any British Government will at any time sanction the introduction of a larger number of nursing sisters to the military hospitals than the trusted superintendents of the Army Nursing Service consider to be desirable for the proper nursing of the patients in the sick wards of our military hospitals. So long as the number of sisters is equal to the nursing requirements of the sick ward population of military hospitals all will be well. The convalescent ward patients are much better left in the charge of male attendants and orderlies. This is the key to the wise solution of this much-debated question.

#### More Independence Necessary.

The oftener one visits a military hospital, especially an important British institution, the more impressed one becomes with the need that exists for a material alteration in the *esprit* of the medical administration of these institutions. For years an agitation has been proceeding, which is now happily ended, in favour of giving greater privileges and a better position to the members of the medical department of our army. It has been pointed out over and over again that the existence of the old system, whereby the medical officer received less than his due, has resulted to a large extent in keeping out of the army the class of men whom it is desirable to induce to enter its medical service. This circumstance must have had a material effect upon the *personnel* of the senior members of the Army Medical Service, which is largely governed by tradition; and that tradition unfortunately tends to kill enterprise and break the spirit of the younger men who, under happier conditions, are now entering it. It is impossible to go over a military hospital, with one or two exceptions, and look carefully into the details of management, without becoming aware that these institutions are behind the civil hospitals in many matters of administration. It has unfortunately been true of more than one of our large military hospitals that there have at times been difficulties in getting the necessary money to make the buildings attractive in appearance, and the wards reasonably comfortable and bright for the reception of the sick. Those difficulties, we have reason to know, would have been materially less, and might have been much more readily overcome, had there been greater energy and courage exhibited on the part of the army medical officers in charge of these institutions. It is a great mistake for a senior officer to discourage his junior, when the latter is a man of parts, working in a hospital under his charge, who from his knowledge of the administration of the civil hospitals and the methods which prevail there, desires to raise the average of efficiency in a particular military hospital. We maintain that before a principal medical officer of a military hospital, or the Medical Director General, lays the blame upon the Government, when there is evidence of an absence of necessary appliances or fittings, or marked defects in the structural and sanitary arrangements of a particular military hospital, he or they should be able to produce documentary evidence that all these matters have been brought forward and insisted upon as necessaries by the principal medical officer with the support of his chief, the Medical Director.

It has been our privilege to visit the military hospitals of several European nations during the last two years, and when we come to compare them with our British military hospitals, and to inquire into the reasons why the latter are in certain respects behind, the more we inquire the more we are convinced that the responsibility in the first instance must be laid to the charge of the representatives of our Army Medical Service.

Why Netley was Neglected.

Lord Lansdowne has set an excellent example to all Secretaries of State for War by paying a visit of inspection to Netley Hospital and making his own independent inquiries as to its deficiencies and the best way of bringing it up to the highest standard of efficiency. It is in our judgment matter for regret that the great improvements which are about to be introduced into Netley Hospital, of which we gave an intimation a few weeks ago, should have been delayed so long, and that they should now in justice be more attributable to the Government than to the Army Medical Department. We have great confidence that the evidence which Lord Lansdowne's minute affords of the desire of the Government through the War Office to cordially co-operate with the Army Medical Department in making every military hospital as complete, as attractive, and as efficient as the best civil hospital, having regard to the essential differences which must always exist between the two types of institution, will put heart into those members of the Army Medical Department who may in future have to do with the military hospitals from time to time, and will secure that henceforth they will actively concern themselves in definitely representing, whenever necessary, that certain expenditure is essential to efficiency, and that they cannot make the military hospital what it ought to be unless prompt attention is paid to their recommendations. A little less sleepy timidity and a good deal more vigorous initiative in the administrative side of their hospital work on the part of military medical officers is due to the nation, the Army, and the personal reputation of the gentlemen immediately concerned. It is so easy to put the blame upon the War Office or the Government, but after Lord Lansdowne's action this excuse cannot be accepted in future, nor will it, we hope, be made again by any member of the Army Medical Department, from the Director-General downwards.

